# Knowledge, attitudes, and practices regarding dengue infection among public sector healthcare providers in Machala, Ecuador

**DOI:** 10.1186/s40794-016-0024-y

**Published:** 2016-06-01

**Authors:** Andrew S. Handel, Efraín Beltrán Ayala, Mercy J. Borbor-Cordova, Abigail G. Fessler, Julia L. Finkelstein, Roberto Xavier Robalino Espinoza, Sadie J. Ryan, Anna M. Stewart-Ibarra

**Affiliations:** 1 Department of Pediatrics, Stony Brook Children’s Hospital, Stony Brook, NY USA; 2 Universidad Técnica de Machala, Machala, Ecuador; 3 Ministerio de Salud Publica, Machala, El Oro Ecuador; 4 Facultad de Ingenieria Maritima, Ciencias Oceanicas y Recursos Naturales, Escuela Superior Politécnica del Litoral (ESPOL), Guayaquil, Guayas Ecuador; 5 Division of Nutritional Sciences, Cornell University, Ithaca, NY USA; 6 Department of Geography, University of Florida, Gainesville, FL USA; 7 Emerging Pathogens Institute, University of Florida, Gainesvillee, fl USA; 8 Center for Global Health and Translational Science, State University of New York Upstate Medical University, Syracuse, NY USA

**Keywords:** Dengue fever, KAP survey, Ecuador, Medical practitioners

## Abstract

**Background:**

Dengue fever is a rapidly emerging infection throughout the tropics and subtropics with extensive public health burden. Adequate training of healthcare providers is crucial to reducing infection incidence through patient education and collaboration with public health authorities. We examined how public sector healthcare providers in a dengue-endemic region of Ecuador view and manage dengue infections, with a focus on the 2009 World Health Organization (WHO) Dengue Guidelines.

**Methods:**

A 37-item questionnaire of dengue knowledge, attitudes, and practices was developed and administered to dengue healthcare providers in Machala, Ecuador. Survey focus areas included: “Demographics,” “Infection and Prevention of Dengue,” “Dengue Diagnosis and the WHO Dengue Guide,” “Laboratory Testing,” “Treatment of Dengue,” and “Opinions Regarding Dengue.”

**Results:**

A total of 76 healthcare providers participated in this study, of which 82 % were medical doctors and 14 % were nurses. Fifty-eight percent of healthcare professionals practiced in ambulatory clinics and 34 % worked in a hospital. Eighty-nine percent of respondents were familiar with the 2009 WHO Dengue Guidelines, and, within that group, 97 % reported that the WHO Dengue Guide was helpful in dengue diagnosis and clinical management. Knowledge gaps identified included *Aedes aegypti* mosquito feeding habits and dengue epidemiology. Individuals with greater dengue-related knowledge were more likely to consider dengue a major health problem. Only 22 % of respondents correctly reported that patients with comorbidities and dengue without warning signs require hospital admission, and 25 % of providers reported never admitting patients with dengue to the hospital. Twenty percent of providers reported rarely (≤25 % of cases) obtaining laboratory confirmation of dengue infection. Providers reported patient presumptive self-medication as an ongoing problem. Thirty-one percent of healthcare providers reported inadequate access to resources needed to diagnose and treat dengue.

**Conclusion:**

Participants demonstrated a high level of knowledge of dengue symptoms and treatment, but additional training regarding prevention, diagnosis, and admission criteria is needed. Interventions should not only focus on increasing knowledge, but also encourage review of the WHO Dengue Guidelines, avoidance of presumptive self-medication, and recognition of dengue as a major health problem. This study provided an assessment tool that effectively captured healthcare providers’ knowledge and identified critical gaps in practice.

**Electronic supplementary material:**

The online version of this article (doi:10.1186/s40794-016-0024-y) contains supplementary material, which is available to authorized users.

## Background

Dengue virus infection is a major cause of morbidity, mortality, and economic hardship in the tropics and subtropics [1, 2]. Infection occurs when one of four dengue virus serotypes (DENV 1–4) are transmitted to humans by *Aedes* sp. (primarily *Aedes aegypti*) mosquitoes [1]. Dengue infection may cause fever, headache, abdominal pain, rash, muscle aches, and bone pain (hence ‘break-bone fever’). Infection with additional dengue serotypes increases the risk of hemorrhagic disease, resulting in severe mucosal and gastrointestinal bleeding, hypovolemia, and potentially death [1]. It is crucial that healthcare professionals are able to accurately diagnose, monitor, treat, and hospitalize patients infected with dengue fever.

Latin America has seen a surge of dengue infections since the 1980s, increasing the need for physicians skilled in managing dengue. From 2010 to 2014, an average of 1.5 million cases per year were reported in the Americas [3], although total case estimates are higher due to underreporting [2]. Díaz-Quijano et al. [4] estimated that dengue-related mortality rates have tripled every decade in Latin America since dengue became endemic in the 1980s. The economic burden of dengue fever is also tremendous: the estimated median cost of dengue treatment in the Americas is US$472 per ambulatory case (72.9 % of cases) and US$1,227 per hospitalized case [5]. The total economic impact of dengue in the Americas was estimated at US$2.1 billion per year (2000–2007 estimate; range US$1–4 billion) [5], underscoring the significant economic burden of dengue fever infection and the need to improve interventions.

Understanding how clinicians manage suspected cases of dengue is crucial to improving patient outcomes. In 2009, the World Health Organization (WHO) revised its classification system of dengue severity [6]. The central aim of the new scheme is to improve clinical outcomes by identifying patients at highest risk of mortality who may require therapeutic interventions. However, acceptance and incorporation of these recommendations has varied considerably since publication, with ongoing debate regarding the utility of each classification scheme [7, 8]. Recent investigations of the WHO Dengue Guidelines are promising. Prasad et al. [9] compared the sensitivity of the 2009 and 1997 WHO guidelines in identifying the severity of dengue infection among 56 patients who tested positive for dengue infection in northern India. The study found that, when compared to the ‘gold standard’ of actual level of medical intervention provided (i.e. outpatient versus inpatient treatment), the 2009 WHO classification system had 98.0 % sensitivity, compared to 24.8 % sensitivity using the 1997 system. In an analysis of 1,962 cases reviewed from 18 countries, Barniol et al. [10] found that 13.7 % of cases could not be classified using the 1997 WHO classification system, compared to 1.6 % using the 2009 WHO classification system. As the debate over dengue classification continues, it is critical to understand how clinicians interpret and apply the guidelines in clinical practice.

Previous research has focused on how community members view dengue infections; however, there have been few attempts to date to better understand the perspectives of clinicians. These studies have been conducted mainly in Asia [9, 11–15], with a single study performed in Puerto Rico [16]; to our knowledge, no studies to date have been conducted in Central or South America. Results have varied considerably across these studies. In a study of Sri Lankan practitioners [11], Kularatne et al. report significant disagreement among physicians over the utility of treating dengue with steroids, antibiotics, and platelet transfusions. Lee et al. [12] noted that clinical practice varied significantly by practice setting, as physicians practicing in private practice were more likely to refer patients with dengue to the hospital and to utilize dengue PCR testing (vs. serology), compared to physicians practicing at public clinics. Thaver et al. [13] conducted a knowledge-based assessment in Pakistan and found that practitioners had a stronger understanding of dengue pathophysiology than clinical diagnosis and treatment. Together, these studies provide evidence that clinical practice varies by region and over time, making it crucial to understand local, current practices for dengue management when identifying areas of potential improvement.

As the epidemiology of dengue has evolved over the past century, so have healthcare systems’ strategies to reduce infection rates. Healthcare providers who interact directly with patients have an important role in both treating and preventing the spread of dengue. This study was conducted to assess the knowledge, attitudes, and practices regarding dengue infection among healthcare providers in a dengue-endemic city in Ecuador. We also assessed familiarity with the 2010 Pan American Health Organization’s (PAHO) Spanish translation [17] of the 2009 WHO Dengue Guidelines, and how these guidelines influenced their clinical practice, providing important information to help guide future interventions.

## Methods

### Study site and study population

We conducted a study of the knowledge, attitudes, and practices associated with dengue infection among healthcare providers practicing in Machala, Ecuador, from December, 2013 through December, 2014. Machala is an urban coastal city located in El Oro Province, Ecuador (3.2667°S, 79.9667°W, altitude 6 m, population 245,972), and has been well-described as hyper-endemic for dengue fever (DENV 1–4) [18, 19]. Over a five year period (2010 to 2014), 72,060 cases of dengue were reported in Ecuador, with an annual average of 14,412 cases [20]. This study is part of an ongoing collaboration with the Ministry of Health to strengthen dengue surveillance capacities, with the aim of studying public sector healthcare providers; private physicians were therefore not included in our study. The Ecuadorian Ministry of Health previously collaborated with the Pan American Health Organization (PAHO) to translate the 2009 WHO Dengue Guidelines into a 2010 Spanish version of the guidelines [17], which was distributed throughout Machala and serves as a focal point of our study.

Physicians and nurses were recruited as the study population because they serve as the frontline healthcare workers for diagnosis and treatment of dengue and other febrile illnesses. Healthcare providers in Machala include primary care providers working in local healthcare clinics (Centros de Salud) and tertiary care providers practicing in public and private hospitals, including emergency care physicians, hospitalists, and subspecialists. The public health system requires that individuals visit a single assigned Centro de Salud prior to referral to hospital subspecialists. These clinics provide care free of charge. Private clinics were not included in this study. It is common for Ecuadorians to view hospital care as superior to ambulatory clinics, leading some patients to seek primary care in the Emergency Department.

### Participant recruitment

Two methods of recruitment were utilized in this study. Participants from the public health sector were recruited at dengue management training conferences in Machala, with survey distribution prior to the educational session. These trainings were conducted in collaboration with and sponsored by the Ecuadorian Ministry of Health and the Global Emerging Infections Surveillance and Response System (GEIS, a division of the United States Armed Forces Health Surveillance Center), with the goal of improving recognition of dengue infection and awareness of the World Health Organization’s Clinical Manual of Dengue. These individuals were recruited for the training sessions as they play key roles in dengue management. The second form of recruitment involved visits to the Ministry of Health public health clinics and to the Teófilo Dávila Hospital, the reference hospital for the province of El Oro.

### Questionnaire development

We developed a 37-item questionnaire, with the goal of evaluating the knowledge, attitudes, and practices associated with dengue infection among healthcare providers. Information regarding dengue infection was based on the World Health Organization’s Clinical Manual of Dengue, with a subset of questions on local dengue epidemiology based on peer-reviewed sources [3, 13, 18]. The questionnaire comprised of the following sections: “Demographics,” “Infection and Prevention of Dengue,” “Dengue Diagnosis and the WHO Guide,” “Laboratory Testing,” “Treatment of Dengue,” and “Opinions Regarding Dengue” (See Additional file 1: Appendice A1 for English and Additional file 2: Appendice A2 for Spanish versions of the survey instrument). The survey was piloted through face-to-face interviews with physicians in Machala prior to conducting the full study.

### Data analysis

Survey responses were analyzed using R (Version 3.1.2). Descriptive statistics (e.g. means, medians, frequency distributions) were calculated. A Cumulative Knowledge Score (CKS) was calculated as an aggregate of all knowledge-based questions (See questions in Tables 2 and 3). Correct answers received one point and incorrect answers received zero points, for a maximum possible score of 14 points. Questions requiring participants to select multiple correct answer choices were given one point per correct answer selected. A Clinical Scenario Score (CSS) was similarly developed from three clinical questions, with a maximum score of three points (See Table 3). These same clinical questions were included in the CKS. Bivariate Pearson Correlations (r) were conducted to assess whether the CSS and CKS were associated with awareness and/or support of WHO clinical guidelines, prior training, years of experience or number of patients treated, and region of medical practice. We also examined whether dengue risk perceptions were associated with support for the WHO dengue guidelines, and the proportion of patients referred for dengue laboratory testing or hospital admission. The questions were grouped by dependent variable, and a Bonferroni correction was used for multiple comparisons. The alpha level was set at 0.05 (i.e., values of *p* <0.05 were considered statistically significant).

Closed-ended questions using a Likert scale and open-ended questions were used to assess doctor and patient perceptions of dengue (See Tables 5, 6 and 7). The frequencies of these themes were tabulated, and for each theme, and the average scores from the Likert scale were used to identify themes that associated with greater risk perceptions.

## Results/Discussion

In this study, several common themes emerged; healthcare providers reported:

1. High use and awareness of the 2009 WHO Dengue Management Guidelines.
2. High level of knowledge regarding dengue signs and symptoms, but demonstrated significant knowledge gaps regarding dengue epidemiology and prevention.
3. Limited knowledge of WHO-recommended criteria for dengue hospital admission, and under-utilization of confirmatory laboratory tests.
4. High level of concern regarding the burden of dengue in Machala; and a lack of training and basic tools needed to adequately diagnose and manage dengue infections.
5. High levels of presumptive self-medication and delay in seeking medical attention among patients with dengue fever.

This study assessed the knowledge, attitudes and practices of local healthcare providers in dengue management. This study was restricted to one group of healthcare practitioners in Machala at one point in time, and accordingly, the small sample size of available providers within Machala may limit generalizability of findings. Additionally, data collected were self-reported, limiting our ability to assess healthcare practices and causal inference. However, this study captures useful information from a community with a high burden of dengue, and this assessment framework can inform dengue management in other settings.

### Demographics

A total of 76 healthcare providers involved in dengue care and treatment in Machala, Ecuador, participated in the study. Demographic information is presented in Table 1. Surveys were administered to participants during visits to their offices or at training events, resulting in a 100 % response rate. Forty-one percent of participants were male and 59 % were female. Ages ranged from under 30 years old to less than 70 years old, with a median age group of 41 to 50 years old and a median of 10 to 14 years of healthcare experience. Participants consisted of physicians (82 %), nurses (14 %), and other healthcare professionals (4 %). There are 93 physicians working in the public health sector in Machala including 63 doctors at 17 public health clinics, 15 in the central hospital, and 15 in the social security hospital, giving an inclusion rate of 67 % of all potential physician subjects in the city. Healthcare providers worked primarily in public health clinics (58 %) and the Teófilo Dávila Hospital (34 %).

**Table 1:** Characteristics of Study Participants (*n* = 76)

Category	Response selected	*n* (%)
Gender	Male	31 (41 %)
	Female	45 (59 %)
Age (years)	<30	24 (32 %)
	31–40	10 (14 %)
	41–50	17 (23 %)
	51–60	17 (23 %)
	61–70	6 (8 %)
	>70	0 (0 %)
Medical role	Doctor	62 (82 %)
	Nurse	11 (14 %)
	Other	3 (4 %)
Years of Medical experience	<1	6 (8 %)
	1–4	26 (34 %)
	5–9	5 (7 %)
	10–14	7 (9 %)
	15–19	10 (13 %)
	>19	22 (29 %)
Practice setting (*n* = 74)	Community Health Center (Subcentro de Salud)	43 (58 %)
	Hospital	25 (34 %)
	Diagnostic Laboratory	2 (3 %)
	Other	4 (5 %)

### Healthcare provider views of dengue burden

As seen in Table 2, healthcare providers in Machala were concerned with dengue infections, with 89 % of participants agreeing that it is a “major problem for my patient population”. Of those in agreement, the majority reported that dengue is a significant threat because the virus is endemic to the region and has the potential to cause high morbidity. One participant reported that dengue may cause “the deterioration of the [individual], family and community health”. The majority of respondents (78 %) also agreed with the statement, “My patients feel that dengue infection is a major problem for their health,” with 34 % citing health complications and mortality as the major concerns in the general population. Seventeen percent of participants suggested that public health measures within the city, including disease prevention efforts, local infrastructure, and education were inadequate for controlling disease transmission. A small subset (6 %) of providers reported that dengue is not a major problem because the region already has effective prevention and treatment interventions in place. A similar proportion of providers also felt that a dengue diagnosis creates an unnecessary sense of fear among patients.

**Table 2:** Physician responses to the statement “I think that dengue is a major problem for my patient population” (*n* = 71)

Categorical responses	Open-ended responses
Agree or Strongly Agree (*n* = 63, 88 %)	The region is an endemic zone
	Dengue has a high morbidity
	There is a lack of preventative measures
	Patients self-medicate
	There exists poor infrastructure
	Dengue poses a high risk to others
	There is a lack of education about dengue
	There is a lack of social consciousness regarding dengue
Neutral (*n* = 4, 6 %)	There is adequate education about dengue
Disagree or Strongly Disagree (*n* = 4, 6 %)	Good preventative measures are in place
	Good medical attention is available

### Providers’ views of the community response to dengue and self-medication

The majority (76 %) of healthcare providers perceived that patients exhibiting symptoms of dengue would seek attention at a healthcare facility. An equal proportion also reported that patients are aware of the steps needed to prevent dengue infection (See Tables 2, 3 and 4 for physician attitudes toward dengue); of those who agreed with this statement, 30 % suggested that public health awareness campaigns were successful. For example, one participant reported, “due to constant [educational] campaigns, [patients] know to seek out medical help before they develop alarms signs”.

**Table 3:** Physician responses to the statement “My patients feel that dengue infection is a major problem for their health” (*n* = 68)

Categorical responses	Open-ended responses
Agree or Strongly Agree (*n* = 53, 78 %)	Dengue has a high morbidity
	Dengue is considered an alarming diagnosis
	Dengue decreases economic productivity
	Dengue is difficult to detect
	The environment is conducive to disease transmission
	Dengue poses a high risk to others
Neutral (*n* = 7, 10 %)	Adequate medical attention is available
Disagree or Strongly Disagree (*n* = 8, 12 %)	Patients believe self-medication is adequate
	There is a lack of education about dengue

**Table 4:** Physician responses to the statement “In my experience, a member of the community who exhibits dengue symptoms will seek medical attention” (*n* = 71)

Categorical responses	Open-ended responses
Agree or Strongly Agree (*n* = 54, 76 %)	Patients believe that dengue has a high morbidity if untreated
	Dengue symptoms are severe
	Patients want to prevent complications
	There is adequate education about dengue
	There is easy access to medical attention
Neutral (*n* = 7, 10 %)	Some patients will seek attention while others self-medicate
Disagree or Strongly Disagree (*n* = 10, 14 %)	Patients do not seek medical attention until complications develop
	Medical care is delayed by self-treatment

Seventeen percent of providers reported that upon symptom onset, many patients “turn to self-medication and do not seek out professional help”. Previous studies have reported that communities in the urban periphery, and particularly men, report self-medicating to treat dengue [21]. This tendency to self-medicate can result in greater dengue morbidity and mortality due to lack of clinical management, and has the potential to increase community susceptibility to other diseases by promoting bacterial resistance to over-prescribed antibiotics.

### Clinical scenario scores and cumulative knowledge scores

The Cumulative Knowledge Score analysis results are presented in Tables 5 and 6 (Table 6 consists of the Clinical Scenario subset of questions). The mean Cumulative Knowledge Score was 10.5 of 14 possible points (SD ± 1.73). Using a Bonferroni correction, the statistical significance level for CKS was determined to be *p* <0.01. The Cumulative Knowledge Score correlated positively with: 1) reporting familiarity with the WHO Dengue Guide (*r* = 0.427, *p* <0.01), 2) agreeing with the statement “I believe that dengue is a major problem for my patient population” (*r* = 0.433, *p* <0.01), and 3) agreeing to the statement “My patients feel that dengue infection is a major problem for their health” (*r* = 0.282, *p* <0.01). Notably, having previous dengue training was not significantly correlated with the CKS (*p* = 0.225). These associations provide evidence of the interrelatedness of a practitioner’s knowledge, patient care, and concern for dengue infection. Clinician education must not only focus on basic knowledge, but also emphasize dengue’s burden on individual health and communities.

**Table 5:** Knowledge-Based Questions (*n* = 76)

Question	Correct response	*n* (%) with correct response
1. How is dengue spread?	Aedes mosquito	75 (99 %)
2. At what time of day are people most likely to be infected by dengue?	Any answer other than “Night”	57 (75 %)
3. Which of the dengue serotypes have been found in Ecuador?	DENV 1–4 are all present	
- **Note:** 0.25 point given per correct answer, with a total of 1 point available	DENV 1	54 (71 %)
	DENV 2	54 (71 %)
	DENV 3	48 (63 %)
	DENV 4	34 (45 %)
4. What advice do you give your patients to prevent dengue infection? - **Note**: Question is worth a total of 2 points - 0.25 point given per correct answer, with a maximum of 1 point. Column to the right indicates the n (%) of respondents receiving **-** 1 point given for not selecting “Take Paracetamol”	1. Frequently change the water in flower vases	66 (87 %)
	2. Remove containers that accumulate clean water	69 (91 %)
	3. Eliminate tanks or puddles with stagnant water	55 (72 %)
	4. Keep drinking water containers (cisterns, tanks) tightly closed	67 (88 %)
	Did not select “Take Paracetamol”	54 (71 %)
5. Which group of patients should be hospitalized? - **Note:** Each response is worth 1 point. Question is worth a total of 4 points - If the answer is correctly selected, the respondent gains 1 point - If the answer is correctly left blank, the respondent gains 1 point - Responses that are correct are marked here as (T) and if incorrect are marked as (F)	1. Dengue without warning signs (F)	76 (100 %)
	2. Dengue without warning signs but with comorbidities (T)	17 (22 %)
	3. Dengue with warning signs (T)	59 (78 %)
	4. Severe dengue (T)	58 (76 %)
	Percent answering all 4 correctly	15 (20 %)
6. According to the WHO’s 2010 Clinical Management of Dengue guidebook, what signs and symptoms can be used to identify an infection of dengue without alarm signs?	Correct Responses	*n* (%) selecting response
- **Note:** Question is worth 1 point - Each response is worth 1/19 point, which is given for either correctly selecting a true response or correctly leaving a false response blank	Headache	59 (78 %)
	Muscle pain	60 (79 %)
	Retro-orbital pain	62 (82 %)
	Positive tourniquet test	45 (59 %)
	Fever/subjective warmth	64 (84 %)
	Petechial rash	33 (43 %)
	Vomit	25 (33 %)
	Incorrect	
	Ascites	1 (1 %)
	Constipation	5 (7 %)
	Diarrhea	10 (13 %)
	Dyspnea	3 (4 %)
	Dysuria	2 (3 %)
	Chest pain	1 (1 %)
	Edema	2 (3 %)
	Icterus	1 (1 %)
	Lymphadenitis	3 (4 %)
	Nasal secretions	11 (14 %)
	Persistent cough	3 (4 %)
	Thrombocytopenia	15 (22 %)
7. Select any the treatments you could use in a patient suspected to have dengue	Oral Hydration	70 (92 %)
- **Note**: Question is worth 1 point - 0.5 points given for hydration (either oral and/or IV) and 0.5 points given for paracetamol. Recipient is given 0 points if anti-bacterial or anti-viral medication is selected	IV Hydration	12 (16 %)
	Paracetamol	71 (93 %)
	Anti-bacterial	1 (1 %)
	Anti-viral	1 (1 %)
	Any of the following (listed individually in survey): Aspirin, NSAIDs/Steroids/Immunosuppressants (methotrexate, cyclosporine, etc.)/Opioids/Platelets/Plasma/Whole blood transfusion	0 (0 %)

**Note:** One point given per question, unless otherwise specified

**Table 6:** Clinical Knowledge Questions

Question	Response Selected	*n* (%)
1. An 8-year old male patient presents to your office with a 4 day history of fever, nausea, vomiting three times per day, and joint aches. He is accompanied by his mother, who reports that he has been less active over the past few days and seems to be getting more uncomfortable. You note the following abnormalities on physical exam: The patient has bleeding of the oral mucosa, a palpable mass on the right side 2 cm below the ribs, and winces when you palpate his abdomen. You do not observe fluid in the abdomen or difficulty breathing. Based on current WHO guidelines, this patient is best classified as:	(*n* = 73)	
	Dengue fever	0 (0 %)
	Dengue hemorrhagic fever	5 (7 %)
	Dengue shock syndrome	0 (0 %)
	Dengue without warning signs	2 (3 %)
	Dengue with warning signs (T)	61 (83 %)
	Severe dengue	5 (7 %)
2. A 5-year-old girl patient presents to your office with a few days of fever and a distended, painful abdomen. Her mother states that she has been less active over the past 3 days. It is currently February and you have seen six patients in the past 3 weeks with dengue infections. The best course of action in managing this patient is to:	(*n* = 73)	
	Order dengue lab tests, tell the patient to get rest at home, and ask the patient to return to your office in 24 h	10 (14 %)
	Order dengue lab tests and admit the patient to the hospital for 24 h of observation (T)	54 (74 %)
	Order dengue lab tests and admit the patient to the Intensive Care Unit for close monitoring and access to emergency care	9 (12 %)
3. A 27-year-old male patient presents to your office in February with two days of fever and complaints of muscle aches. He notes that he has had three episodes of non-bloody vomiting in the past two days. The patient notes that his younger sister has similar symptoms. You recall hearing numerous reports of dengue infection during the last month. The best course of action in managing this patient is to:	(*n* = 71)	
	Order dengue lab tests, tell the patient to get rest at home, and ask the patient to return to your office in 24 h (T)	52 (73 %)
	Order dengue lab tests and admit the patient to the hospital for 24 h of observation	19 (27 %)
	Order dengue lab tests and admit the patient to the ICU for close monitoring and access to emergency care	0 (0 %)

Note: Each question is worth 1 point. (T) if placed next to the correct response

The Clinical Scenario Score analysis results are presented in Table 6. The mean Clinical Scenario Score was 2.1 of 3 potential points (Table 6). Using a Bonferroni correction, the statistical significance level for CSS was determined to be *p* <0.0125. A higher CSS was correlated with the following responses: 1) reporting familiarity with WHO Dengue Guidelines (*r* = 0.326, *p* <0.01), 2) agreeing with the statement “I believe that dengue is a major problem for my patient” (*r* = 0.37, *p* <0.01), 3) agreeing with the statement “I am fully trained to manage a patient with an infection of dengue without warning signs,” (*r* = 0.383, *p* <0.01), and 4) agreeing with the statement “In my experience, a community member who has dengue symptoms will seek medical attention” (*r* = 0.453, *p* <0.01). Higher CSS was also associated with reporting that the WHO Guidelines are helpful, although this was not statistically significant after a Bonferroni correction was applied (*r* = 0.245, *p* <0.05). These findings emphasize the importance of practitioner ‘buy-in’ of dengue’s detrimental impact, as clinical knowledge and concern for dengue infections are strongly associated. Of note, the clinical scenarios comprised a small component (3 of 14 points) of the above-mentioned Cumulative Knowledge Score.

### Providing patients with accurate dengue prevention and treatment guidance

Although study participants demonstrated a high level of understanding of dengue infection signs, symptoms, and treatment, we identified specific gaps in knowledge of dengue prevention and epidemiology. A total of 29 % of participants incorrectly selected “take paracetamol” as a method for preventing dengue infection (Table 5). Although it is possible that some participants misinterpreted this question as asking which medications may help manage dengue, the survey clearly asked how dengue may be *prevented*, indicating a misconception of prevention strategies. In addition, 25 % incorrectly selected “night time” as the most likely feeding time for *Aedes* mosquitoes. Similar findings have been documented elsewhere: Huang et al. [14] found that only 14.4 % of Taiwanese providers correctly identified *Aedes* mosquito feeding habits, compared to 82.8 % who correctly identified *Anopheles* mosquito feeding habits. When participants were asked which dengue virus serotypes are found in Ecuador, only 38 % correctly answered all four serotypes (DENV 1–4). Ho [15] also found limited knowledge of dengue epidemiology among healthcare providers in Taiwan, with only 47.7 % correctly responding that dengue is endemic in that country. These misunderstandings may lead healthcare providers to give patients incorrect, clinically significant advice. It is critical to target specific local misconceptions of dengue prevention and transmission through training of medical professionals, in order to reduce the burden of dengue.

### Confusion regarding hospital admission criteria

Clinicians indicated confusion when developing appropriate dengue treatment plans for their patients. When healthcare professionals were asked which groups of patients with dengue require hospital admission, only 22 % correctly stated that patients with “dengue without warning signs but with comorbidities” require hospital admission (Table 5). The 2009 WHO Dengue Guidelines provide specific recommendations for appropriate clinical observation based on a patient’s risk of significant morbidity [7]. These guidelines state that any patient with a comorbidity (e.g. diabetes mellitus, obesity, risk of hemorrhage such as peptic ulcer disease) should be admitted to a hospital during a dengue infection, regardless of the severity of infection. Additionally, only 45 % of participants correctly responded to all three clinical scenarios (Table 6), demonstrating knowledge gaps of patient admission criteria.

Hospital admission rates for dengue infection vary considerably between regions globally. For example, Tomashek et al. [16] found that only 31 % of Puerto Rican medical providers used hospital admission criteria consistent with the 1997 WHO Dengue Guidelines. Conversely, Lee et al. [12] reported that one-third of providers in Singapore “always” or “often” admitted patients with suspected dengue, regardless of infection severity. Globally, it is estimated that less than 5 % of patients infected with dengue will develop severe disease [22], and WHO recommends that patients who do not meet criteria for hospitalization have frequent office follow-up [7]. This is particularly important in resource-limited settings. Patients with comorbidities who are not admitted to hospitals may have worse clinical outcomes, underscoring the need for close monitoring of this patient population.

### Diagnostic testing: under-utilization and inadequate resources

Study participants indicated suboptimal use of confirmatory diagnostic laboratory tests when dengue infection was suspected (Table 7). As appropriate in a region with many acute febrile illnesses with similar clinical presentations as dengue, 61 % of healthcare providers reported referring all patients with suspected dengue infection for laboratory test confirmation. However, 20 % of participants reported referring patients for confirmatory laboratory tests 25 % of the time or less. As these patients may actually be infected with other febrile illnesses such as leptospirosis, malaria, or chikungunya, laboratory confirmation is crucial for differential diagnosis and to inform appropriate medical interventions. It is important to note that 14 % of providers reported inadequate access to diagnostic testing for dengue (Table 7). Additionally, providers who agreed with the statement “I am fully trained to manage a patient with an infection of dengue without warning signs” referred a higher percentage of their patients for laboratory testing (*r* = 0.345, *p* <0.01), compared to those who disagreed with this statement. This may signal one of two possibilities: clinical confidence is increased with better access to diagnostic testing, or providers who report greater confidence in their clinical training refer more patients for confirmatory laboratory tests. Access to dengue diagnostic testing remains a key issue in this context.

**Table 7:** Practice-Based Questions

Question	Response selected	*n* (%)
Approximately how many patients do you see per week? (*n* = 60)	0	3 (5 %)
	1–49	6 (10 %)
	50–99	28 (46.7 %)
	100–149	16 (26.7 %)
	>150	7 (11.7 %)
Are you familiar with the WHO’s 2010 Clinical Management of Dengue guidelines?	Yes	67 (89 %)
	No	8 (11 %)
Do you feel that the WHO’s Dengue guidelines help in managing dengue?	Yes	64 (97 %)
	No	2 (3 %)
Of those patients who you suspect have dengue fever, approximately what percentage do you refer to a lab for diagnostic testing?	0 % of patients	1 (1 %)
	10 % of patients	10 (15 %)
	25 % of patients	3 (4 %)
	50 % of patients	8 (12 %)
	75 % of patients	5 (7 %)
	100 % of patients	40 (61 %)
Do your patients ever use a private lab without a referral?	Yes	31 (47 %)
	No	35 (53 %)
Approximately what percentage of patients with dengue fever do you refer to the hospital for additional medical treatment?	0	14 (26 %)
	<10 %	31 (57 %)
	25 %	1 (2 %)
	50 %	6 (11 %)
	75 %	1 (2 %)
	100 %	1 (2 %)
Do you feel you have adequate resources to treat your patients when they have dengue?	Yes	48 (69 %)
	No	22 (31 %)
If you said ‘No’ to the previous question, what are you lacking? - Note: Percentages given as n/22, based on previous question - Note: Subjects may select multiple options	Sufficient training	7 (32 %)
	Medication needed to treat	9 (41 %)
	Instruments needed to treat	8 (36 %)
	Access to lab tools	10 (45 %)

**Note:** Percentages given do not include respondents who did not answer the question

Healthcare providers were asked about availability and access to a variety of resources for dengue diagnosis and treatment. A total of 31 % of providers reported having inadequate resources (See Table 7 for specific resources). There were no significant correlations between reported lack of resources for dengue diagnosis and treatment and insufficient training, Cumulative Knowledge Scores, treatments used, or other items from this survey. Identifying the impact of resource deficiencies is difficult to assess from the data collected, as no discernible differences in knowledge, attitudes, or practices were identified in this study. Further investigation of availability and access to resources for dengue diagnosis and treatment, and how they influence daily clinical practice is needed.

### Awareness and Implementation of the WHO dengue guidelines

Awareness of the 2009 WHO Dengue Guidelines was high, with 89 % of participants reporting previous knowledge of the guidelines. Of these respondents, 97 % reported that these guidelines were helpful. This finding is in contrast to Kularatne’s study of Sri Lankan practitioners [13], in which only 45 % of practitioners reported using the WHO Dengue Guidelines. However, Kularatne’s study was conducted prior to the current version of the WHO Dengue Guidelines, and may be more related to local medical practices and training.

### Impact of practice setting

In this study, there were no significant differences in reported knowledge, attitudes, and practice, between healthcare providers practicing in a hospital versus ambulatory settings, including familiarity with the 2009 WHO Dengue Guidelines, reporting that these guidelines were helpful, or overall dengue knowledge (*p* >0.05). Previous studies have indicated that practice settings can influence clinical management of dengue fever. Ho et al. [15] found that healthcare providers practicing at Taiwanese medical centers (i.e. medical school-affiliated hospitals at the highest accreditation level) had significantly different levels of knowledge, compared to providers at non-medical centers. In order for dengue interventions to be most effective in hyper-endemic regions, healthcare providers of all types and at all settings must receive adequate training and guidance, and differences in knowledge, attitudes, and practice by setting should continue to be assessed.

## Conclusion

Findings from this study provide important insights into medical practitioner knowledge, attitudes, and practices associated with dengue fever in a resource-limited endemic region. These findings highlight several strategies to improve diagnosis and clinical management of dengue infections in this region. A strong healthcare policy begins with accurate information, which can best be obtained and disseminated through close collaboration between the public, primary healthcare providers, health educators, and the public health sector.

- Healthcare providers should receive continuous education about dengue prevention, transmission, and high-risk patient populations.
- Providers’ needs should be assessed in future studies, as nearly one-third of participants reported inadequate access to crucial healthcare resources.
- Health providers should educate their patient population about the harms of self-diagnosis and presumptive self-medication.
- Findings demonstrated that those providers who showed the greatest concern of dengue infections were also the most knowledgeable and provided clinical care that more closely aligned with WHO recommendations. Future interventions should therefore provide core dengue information while emphasizing dengue’s impact on health and development.
- Periodic reassessment of the local knowledge, attitudes, and clinical practices will be instrumental to reduce the burden of dengue fever and improve clinical management in high-burden settings.

## Abbreviations

CKS, Cumulative Knowledge Score; CSS, Clinical Scenario Score; PAHO, Pan American Health Organization; WHO, World Health Organization

## Additional files

Additional file 1: Appendix A1: Knowledge, Attitudes, and Practices of Dengue Survey – English Version. (DOC 49 kb)

Additional file 2: Appendix A2: Knowledge, Attitudes and Practices of Dengue Survey – Spanish Version. (DOC 55 kb)
