# Supplementary material for: Knowledge, attitudes, and practices regarding dengue infection among public sector healthcare providers in Machala, Ecuador
Source: Trop Dis Travel Med Vaccines. 2016 Jun 1;2:8. doi: 10.1186/s40794-016-0024-y (PMC5531027; doi:10.1186/s40794-016-0024-y)
Supplement: Supplementary file 1 — Appendix A1: Knowledge, Attitudes, and Practices of Dengue Survey – English Version. (DOC 49 kb) [file 40794_2016_24_MOESM1_ESM.doc]

Additional file 1

Appendix A1: Knowledge, Attitudes, and Practices of Dengue Survey – English Version

**Instructions:**  Please place a check mark in the box that best selects your answer choice.

**Section 1: Demographics**

1. What gender are you?
   - Male
   - Female
2. How old are you?
   - Under 30 years
   - 31-40
   - 41-50
   - 51-60
   - 61-70
   - Over 70 years
3. What is your role in the medical community (doctor, nurse, technician, etc.)?

________________________________________________________________

1. If applicable, what is your specialty?
   - Internal Medicine
   - Pediatrics
   - Neurology
   - Surgery
   - Obstetrics/Gynecology
   - Not applicable
   - Other: ________________________________________________
2. If applicable, what is your subspecialty?

______________________________

1. What district of Machala do you work in?

______________________________

1. What is the highest level of education you have completed?
   - Middle School
   - High School
   - University/College
   - Master’s Degree
   - MD
   - PhD
   - Other ________________________
2. How long have you been working in the health field?
   - <1 year
   - 1-4 years
   - 5-9 years
   - 10-14 years
   - 15-19 years
   - 20 or more years
3. What type of a facility do you work in?
   - Community Health Center
   - Hospital
   - Private office/Clinic
   - Diagnostic Laboratory
   - Other: _______________________
4. Have you received training in treating dengue?
   - Yes
   - No

If yes, please describe the training: ________________________________________________________________

11. Approximately how many patients do you see per week?

________________________________

**Section 2: Dengue Infection and Prevention**

1. How is dengue spread?

**Please select all answers that apply.**

- *Aedes aegypti* mosquito bite
- *Anopheles* mosquito bite
- Dirty water
- Bite by lice
- Bite by ticks
- Under-cooked food

1. At what time of day are people most likely to be infected by dengue?

**Please select all answers that apply.**

- Morning
- Noon
- Evening
- Night

1. Which of the dengue serotypes have been found in Ecuador?

**Please select all answers that apply.**

- DENV1
- DENV2
- DENV3
- DENV4

1. In your opinion, are your patients aware of steps to take to prevent dengue?

- Yes
- No

1. What advice do you give your patients to prevent **dengue** infection?

**________________________________ ­­­________________________________**

**Please select all answers that apply.**

- Frequently change the water in flower vases
- In general, healthy people should sleep under bed nets every night
- Remove containers that accumulate clean water (bottles, tires, cans)
- Eliminate tanks or puddles with stagnant water
- Keep drinking water containers (cisterns, tanks) tightly closed
- Keep the house closed
- Request malaria fumigation
- Take paracetamol
- Other: ________________________________________________

**Section 3: Diagnosing Dengue and WHO Guidelines**

1. In your clinical experience, how do you differentiate dengue infection from other causes of illness (malaria, leptospirosis, etc.)?

___________________________________________________________

___________________________________________________________

__________________________________________________________

1. Are you familiar with the WHO’s 2010 Clinical Management of Dengue guidelines?

- Yes
- No (If no, skip to the following section, **Laboratory Testing**)

1. Do you feel that the WHO’s Dengue guidelines help in managing dengue?

- Yes
- No

Please explain your answer choice: ________________________________________________________________________________________________________________________________________________

1. Which group of groups of patient should be hospitalized?

- Dengue without warning signs
- Dengue without warning signs but with comorbidities
- Dengue with warning signs
- Severe dengue

1. According to the World Health Organization’s 2010 Clinical Management of Dengue guidebook, what signs and symptoms can be used to identify an infection of dengue without alarm signs? **Please select all answers that apply.**

- Ascites
- Constipation
- Headache
- Diarrhea
- Dyspnea
- Dysuria
- Chest pain
- Muscle pain
- Retro-orbital pain
- Edema
- Positive tourniquet test
- Fever/ subjective warmth
- Icterus
- Lymphadenitis
- Petechial rash
- Nasal secretions
- Persistent cough
- Thrombocytopenia
- Vomit

1. Clinical Scenario: An 8-year old male patient presents to your office with a 4 day history of fever, nausea, vomiting three times per day, and joint aches. He is accompanied by his mother, who reports that he has been less active over the past few days and seems to be getting more uncomfortable. You note the following abnormalities on physical exam: The patient has bleeding of the oral mucosa, a palpable mass on the right side 2 cm below the ribs, and winces when you palpate his abdomen. You do not observe fluid in the abdomen or difficulty breathing. Based on current WHO guidelines, this patient is best classified as:

- Dengue fever
- Dengue hemorrhagic fever
- Dengue shock syndrome
- Dengue without warning signs
- Dengue with warning signs
- Severe dengue

**Section 4: Laboratory Testing**

1. Of those patients who you suspect have **dengue fever**, approximately what percentage do you **refer** **to a lab for diagnostic testing**?

- 0%
- 10%
- 25%
- 50%
- 75%
- 100%

1. What labs do you refer your patients to when you suspect they have a dengue infection?

- INSPI Public Lab
- Private Lab
- Other

1. How long does it take to get laboratory results for diagnostic testing?

**___________________________________**

1. Do your patients ever use a private lab without a referral?

- Yes
- No

1. What dengue diagnostic laboratory tests are available through the INSPI Lab?

**Please select all answers that apply.**

- Basic Metabolic Panel
- Complete Blood Count
- Immunoglobin serology
- NS1 ELISA
- RT-PCR
- Other: ________________________________________________

**Section 5: Dengue Management**

1. Select any the treatments you could use in a patient suspected to have dengue:
   - Aspirin, NSAIDs
   - Steroids
   - Oral Hydration
   - Immunosuppressants (methotrexate, cyclosporine, etc)
   - Opioids
   - Paracetamol
   - Intravenous fluid rehydration
   - Antibacterial treatment
   - Antiviral treatment
   - Platelet transfusion
   - Plasma transfusion
   - Whole blood transfusion
   - Other: __________________________
2. Approximately what percentage of dengue fever patients do you **refer** **to the hospital for additional medical treatment**?
   - 0%
   - 10%
   - 25%
   - 50%
   - 75%
   - 100%
3. What medical criteria do you use when you refer a patient with dengue to the hospital?

________________________________________________________________________________________________________________

1. Do you feel you have adequate resources to treat your patients when they have dengue?
   - Yes
   - No

- If you said ‘No’ to the previous question, what are you lacking? **Please select all answers that apply.**
  - Sufficient training­­
  - Medication needed to treat
  - Instruments needed to treat
  - Access to lab tools
  - Other: ___________________________________________________________

1. 2. A 5-year-old girl patient presents to your office with a few days of fever and a distended, painful abdomen. Her mother states that she has been less active over the past three days. It is currently February and you have seen six patients in the past three weeks with dengue infections. The best course of action in managing this patient is to:
   - Order dengue lab tests, tell the patient to get rest at home, and ask the patient to return to your office in 24 hours
   - Order dengue lab tests and admit the patient to the hospital for 24 hours of observation
   - Order dengue lab tests and admit the patient to the Intensive Care Unit for close monitoring and access to emergency care
2. A 27-year-old male patient presents to your office in February with two days of fever and complaints of muscle aches. He notes that he has had three episodes of non-bloody vomiting in the past two days. The patient notes that his younger sister has similar symptoms. You recall hearing numerous reports of dengue infection during the last month. The best course of action in managing this patient is to:
   - Order dengue lab tests, tell the patient to get rest at home, and ask the patient to return to your office in 24 hours
   - Order dengue lab tests and admit the patient to the hospital for 24 hours of observation
   - Order dengue lab tests and admit the patient to the Intensive Care Unit for close monitoring and access to emergency care

**Section 6: Opinions about Dengue**

Use the following rating scale to answer questions 14-17:

**1 2 3 4 5**

Completely Somewhat Neither agree Somewhat Completely

disagree disagree nor disagree agree agree

­­

1. I believe that dengue is a major problem for my patient population.

Number: _______

Why? ___________________________________________________________________

________________________________________________________________________

1. My patients feel that dengue infection is a major problem for their health.

Number: _______

Why? ___________________________________________________________________

________________________________________________________________________

1. I am fully trained to manage a patient with an infection of dengue without warning signs.

Number: _______

Why? ___________________________________________________________________

________________________________________________________________________

1. In my experience, a community member who has dengue symptoms will seek medical attention.

Number: _______

Why? ___________________________________________________________________

________________________________________________________________________
