# Supplementary material for: Knowledge, attitudes, and practices regarding dengue infection among public sector healthcare providers in Machala, Ecuador
Source: Trop Dis Travel Med Vaccines. 2016 Jun 1;2:8. doi: 10.1186/s40794-016-0024-y (PMC5531027; doi:10.1186/s40794-016-0024-y)
Supplement: Supplementary file 2 — Appendix A2: Knowledge, Attitudes and Practices of Dengue Survey – Spanish Version. (DOC 55 kb) [file 40794_2016_24_MOESM2_ESM.doc]

Additional file 2

Appendix A2: Knowledge, Attitudes and Practices of Dengue Survey – Spanish Version

**Instrucciones:** Por favor, marque con un visto el círculo que mejor seleccione su respuesta.

**Sección 1: Demografía**

1. ¿Cuál es su sexo?
   1. Hombre
   2. Mujer
2. ¿Qué edad tiene?
   - Menos de 30 años
   - 31-40
   - 41-50
   - 51-60
   - 61-70
   - Más de 70 años
3. ¿Cuál es su rol en la comunidad médica (doctor, enfermera, técnico, etc.)?

______________________________

1. Si es que aplica, ¿cuál es su especialidad?
   1. Medicina interna
   2. Pediatría
   3. Neurología
   4. Cirugía
   5. Obstetricia/ Ginecología
   6. No aplica
   7. Otro:

_____________________________

1. Si es que aplica, ¿cuál es su subespecialidad?

______________________________

1. ¿En qué parroquia de Machala trabaja?

______________________________

1. ¿Cuál es el nivel más alto de educación que usted ha completado?
   1. Escuela primaria
   2. Colegio secundario
   3. Universidad
   4. Maestría o Diplomado
   5. Doctor en Medicina
   6. PhD
   7. Otro: _____________________
2. ¿Cuánto tiempo ha estado trabajado en el campo de la salud?
   1. <1 año
   2. 1-4 años
   3. 5-9 años
   4. 10-14 años
   5. 15-19 años
   6. 20 o más
3. ¿En qué tipo de instalación trabaja?
   1. Centro de Salud Comunitario
   2. Hospital
   3. Oficina Privada/Clínica
   4. Laboratorio Diagnóstico
   5. Otro: ___________________
4. ¿Ha recibido capacitación en el tratamiento sobre el dengue?
   1. Sí
   2. No

- Si su respuesta es sí, por favor describir esta capacitación: ________________________________________________________________

1. Aproximadamente, ¿cuántos pacientes ve por semana?

________________________________

**Sección 2: Infección y Prevención del Dengue**

1. ¿Cómo se transmite el dengue?

**Por favor, seleccione todas las respuestas que apliquen.**

- Picadura de mosquito *Aedes aegypti*
- Picadura de Mosquito *Anopheles*
- Agua contaminada
- Picadura de Piojos
- Picadura de Garrapata
- Comida semi cruda

1. ¿A qué hora del día es más probable que la gente sea infectada por el dengue? **Por favor, seleccione todas las respuestas que apliquen.**

- En la mañana
- A las doce del día
- Al atardecer
- En la noche

1. ¿Cuáles de los serotipos del dengue han sido encontrados en el Ecuador?

**Por favor, seleccione todas las respuestas que apliquen.**

- DENV1
- DENV2
- DENV3
- DENV4

1. En su opinión, ¿están sus pacientes al tanto de los pasos a tomar para prevenir el dengue?

- Sí
- No

1. ¿Qué consejo da a sus pacientes para prevenir la enfermedad **del dengue**?

**Por favor, seleccione todas las respuestas que apliquen.**

- Cambiar el agua de los floreros con frecuencia
- En general, la gente sana debe dormir bajo toldo todas las noches
- Eliminar recipientes que acumulen agua limpia (botella, llantas, latas)
- Evite estanques o pozas con agua estancada
- Mantener bien tapados los recipientes de almacenamiento agua (cisternas, tanques)
- Mantener cerrada la casa
- Pida a malaria fumigar
- Tome paracetamol
- Otro: ________________________________________________________

**Sección 3: El Diagnóstico del Dengue y Guías de OMS**

1. En su experiencia de la clínica, ¿cómo diferencia la infección del dengue de otras causas de enfermedad (malaria, leptospirosis, etc.)?

___________________________________________________________

___________________________________________________________

___________________________________________________________

1. Se encuentra familiarizado con el Manejo Clínico del Dengue del manual OMS 2010?

- Sí
- No (Si es que no, avance a la siguiente sección, **Pruebas de Laboratorio**)

1. ¿Piensa que las guías de Dengue de OMS ayudan a manejar el dengue?

- Sí
- No

Por favor, explique la respuesta que ha escogido: __________________________________________________________________________________________________________________________

1. ¿Cuáles grupo o grupos de pacientes deben estar ingresados?

- Dengue sin signos de alarma
- Dengue sin signos de alarma pero con comorbilidades
- Dengue con signos de alarma
- Dengue grave

1. De acuerdo a la clasificación del Manejo Clínico del Dengue 2010 de la Organización Mundial de la Salud, ¿qué signos y síntomas se usan para identificar la infección del dengue sin signos de alarma? **Por favor, seleccione todas las respuestas que apliquen.**

- Ascitis
- Constipación
- Cefalea
- Diarrea
- Disnea
- Disuria
- Dolor de pecho
- Dolores musculares
- Dolor retro-orbicular
- Edema
- Examen positivo de torniquete
- Fiebre/ alza térmica
- Ictericia
- Linfadenitis
- Sarpullido petequial
- Secreción nasal
- Tos persistente
- Trombocitopenia
- Vomito

1. Escenario Clínico: Un paciente masculino de 8 años de edad se presenta en su oficina con un historial de 4 días de fiebre, nausea, vomito tres veces por día, y dolores en sus articulaciones. Lo acompaña su madre, quien reporta que él ha estado menos activo los últimos días y parece estar más incómodo. Usted nota las siguientes anormalidades en el examen físico: el paciente tiene sangrado de la mucosa oral, una masa palpable en el lado derecho 2 cm debajo de las costillas, y hace un gesto de dolor cuando palpa su abdomen. No observa fluidos en el abdomen o dificultad en respirar. Basado en la clasificación del dengue del Manejo Clínico del Dengue 2010 actuales, este paciente es mejor clasificado como:

- Fiebre de dengue
- Fiebre hemorrágica de dengue
- Síndrome de shock de dengue
- Dengue sin signos de alarma
- Dengue con signos de alarma
- Dengue grave

**Sección 4: Pruebas de Laboratorio**

1. De todos aquellos pacientes que usted sospecha tienen **fiebre de dengue**, ¿aproximadamente qué porcentaje usted **refiere a un laboratorio para una prueba diagnóstica**?

- 0%
- 10%
- 25%
- 50%
- 75%
- 100%

1. ¿A qué laboratorios refiere a sus pacientes cuando sospecha que tienen el dengue?

- INSPI Laboratorio Público
- Laboratorio Privado
- Otro: ________________________________________________________________

1. ¿Cuánto tiempo toma para recibir los resultados de laboratorio de la prueba de diagnóstico?

**___________________________________**

1. ¿Sus pacientes utilizan un laboratorio privado sin orden emitida por médica?

- Sí
- No

1. ¿Qué pruebas diagnostica de laboratorio del dengue posee actualmente INSPI? **Por favor, seleccione todas las respuestas que apliquen.**

- Ionograma
- Hemograma
- Serología inmunoglobulina
- NS1 ELISA
- RT-PCR
- Otro: ____________________________________________________

**Sección 5: Tratamiento del Dengue**

1. Seleccione que tratamiento utiliza en paciente sospecha de dengue:
   - Aspirina, AINEs
   - Esteroides
   - Hidratación oral
   - Inmunosupresivos (methotrexate, cyclosporine, etc.)
   - Opioides
   - Paracetamol
   - Rehidratación de fluidos intravenoso
   - Tratamiento anti-bacterial
   - Tratamiento antiviral
   - Transfusión de plaqueta
   - Transfusión de plasma
   - Transfusión de sangre completa
   - Otra: ___________________________________________________
2. Aproximadamente, ¿qué porcentaje de pacientes con fiebre de dengue refiere al hospital para tratamiento médico adicional?
   - 0%
   - <10%
   - 25%
   - 50%
   - 75%
   - 100%
3. ¿Qué criterio medico usa para referir a un paciente con dengue a un hospital?

________________________________________________________________________________________________________________

1. ¿Piensa que tiene los recursos adecuados para tratar a sus pacientes cuando tienen dengue?

- Sí
- No

- Si es que contestón ‘No’ a la pregunta anterior, ¿qué es lo que le falta? **Por favor, seleccione todas las respuestas que apliquen.**

- - Capacitación suficiente
  - Medicamente necesario para el tratamiento
  - Instrumentos necesarios para el tratamiento
  - Acceso a herramientas de laboratorio
  - Otro: ___________________________________________________________________

1. Un paciente femenino de 5 años se presenta en su consultorio con cuatro días de fiebre y un abdomen dilatado y doloroso por dos días. Su madre indica que la paciente ha estado menos activa durante los tres últimos días. Es actualmente febrero y usted ha visto seis pacientes en las últimas tres semanas con infecciones de dengue. El mejor procedimiento en tratar a este paciente es:
   - Ordena exámenes de laboratorio, indica al paciente que descanse en su casa, y pide al paciente que regresa al consultorio a las 24 horas.
   - Ordena exámenes de laboratorio y admite al paciente al hospital para una observación de 24 horas.
   - Ordena exámenes de laboratorio y admite al paciente a la Unidad de Cuidados Intensivos para un monitoreo cercano y acceso a cuidados de emergencia.
2. Un paciente masculino de 27 años se presenta en su consultorio en febrero con dos días de fiebre y quejas de dolores musculares. Nota que él ha tenido tres episodios de vomito sin sangre en los últimos dos días. El paciente nota que su hermana menor tiene síntomas similares. Usted recuerda el haber escuchados numerosos reportes de infección de dengue durante el último mes. El mejor procedimiento en el tratamiento de este paciente es:
   - Ordena exámenes de laboratorio, indica al paciente que descanse en su casa, y pide al paciente que regresa a las 24 horas.
   - Ordena exámenes de laboratorio y admite al paciente al hospital para una observación de 24 horas.
   - Ordena exámenes de laboratorio y admite al paciente a la Unidad de Cuidados Intensivos para un monitoreo cercano y acceso a cuidados de emergencia.

**Sección 6: ­­Opiniones sobre el Dengue**

Utilice la siguiente tabla de calificación para contestar las siguientes preguntas:

**1 2 3 4 5**

Completamente En cierto modo Ni de acuerdo ni En cierto modo Completamente

en desacuerdo en desacuerdo en desacuerdo de acuerdo de acuerdo

­­

Creo que el dengue es un gran problema para mi población de pacientes.

Numero: _______

¿Por qué? _______________________________________________________________

________________________________________________________________________

Mis pacientes sienten que la infección del dengue es un gran problema para su salud.

Numero: _______

¿Por qué? _______________________________________________________________

________________________________________________________________________

Estoy completamente capacitado para manejar a un paciente con una infección del dengue sin signos de alarma.

Numero: _______

¿Por qué? _______________________________________________________________

________________________________________________________________________

De acuerdo a mi experiencia, un miembro de la comunidad que tiene los síntomas del dengue buscará atención médica.

Numero: _______

¿Por qué? _______________________________________________________________

________________________________________________________________________
